# 640. Monthly Review of Burn Resuscitations for Collaborative Quality Improvement

**DOI:** 10.1093/jbcr/irag033.477

**Published:** 2026-04-06

**Authors:** Alexandra M Lacey, Joy Ferasol, Ashley Bergamo

**Affiliations:** Regions Hospital, Saint Paul, MN; Regions Hospital, Saint Paul, MN; Regions Hospital, Saint Paul, MN

## Abstract

**Introduction:**

A Nursing Driven Resuscitation Protocol (NDRP) is utilized in our burn unit for the hourly titration of fluids to patients during their burn resuscitation. We found that our monthly Morbidity and Mortality Conferences were not useful for the granular review of our resuscitations, so we implemented a separate multidisciplinary review of every burn resuscitation. We utilized the continuous improvement process of Identify – Plan – Execute – Review to implement real time changes to improve our patient outcomes during their resuscitations.

**Methods:**

Our Process Improvement coordinator carefully tracked and presented the outcomes of every NDRP over a 18-month period. Each month, the burn multi-disciplinary team reviewed the prior month’s resuscitations. The information was presented in an hour-by-hour analysis of the resuscitation. We also tracked other interventions including time to feeding tube placement, blood glucose assessment, appropriate fluid titration, colloid delivery, and bloodwork assessments.

**Results:**

Any adult patient with a total burn surface area (TBSA) >20% or child with a TBSA >15% was included in our analysis. A total of 33 adults and 7 children were analyzed over an 18-month period. Utilizing the process improvement cycle, multiple issues were identified throughout this time and acted on. First, we noted that fresh frozen plasma (FFP) had highly variable volumes; while patients were receiving the prescribed number of units, the volume varied greatly. We transitioned from prescribing units of plasma to volumes of plasma; this allowed more consistent delivery of the prescribed colloid. Secondly, we also saw an increased rate of acute kidney injuries for certain populations. Given this, the resuscitation threshold was reduced to a TBSA of 15% for patients over 60 and those intoxicated with alcohol or methamphetamine.

**Conclusions:**

This practice of monthly review of NDRPs on an hour-by-hour basis revealed multiple areas for improvement. These were acted on in the continuous improvement process of Identify – Plan – Execute – Review and allowed rapid adjustments to improve patient care.

**Applicability of Research to Practice:**

This quality improvement process demonstrates utilization of the continuous improvement process to actuate real world change in burn resuscitations.

**Funding for the study:**

N/A.

